# The Effect of Genotype Combinations of *Wolbachia* and Its *Drosophila melanogaster* Host on Fertility, Developmental Rate and Heat Stress Resistance of Flies

**DOI:** 10.3390/insects14120928

**Published:** 2023-12-05

**Authors:** Natalya V. Adonyeva, Vadim M. Efimov, Nataly E. Gruntenko

**Affiliations:** 1 Institute of Cytology and Genetics SB RAS, Novosibirsk 630090, Russia; nadon@bionet.nsc.ru (N.V.A.); efimov@bionet.nsc.ru (V.M.E.); 2 Department of Natural Sciences, Novosibirsk State University, Novosibirsk 630090, Russia

**Keywords:** *Wolbachia*, *Drosophila*, fertility, developmental rate, heat stress, viability

## Abstract

**Simple Summary:**

*Wolbachia*, the intracellular symbiont of insects, is of big interest and importance for its numerous effects on the host’s life-history traits. However, the details of *Wolbachia*–host interaction are still not well studied and understood. Here, we present data on the influence of two different *Wolbachia* strains on the life-history traits of two different wild-type *D. melanogaster* lines. The results obtained allow us to assume that the effect of *Wolbachia* on the flies’ life-history traits depends on the genotypes of both the host and the symbiont, but the fact of recent transfer of the symbiont to a new host could also be a factor.

**Abstract:**

The best-known effect of the intracellular bacterium *Wolbachia* is its mostly negative influence on the reproduction of the host. However, there is evidence of a positive influence of *Wolbachia* on the host’s resistance to stress, pathogens, and viruses. Here, we analyzed the effects of two *Wolbachia* strains belonging to wMel and wMelCS genotypes on *D. melanogaster* traits, such as fertility, survival under acute heat stress, and developmental rate. We found that *D. melanogaster* lines under study differ significantly in the above-mentioned characteristics, both when the natural infection was preserved, and when it was eliminated. One of *Wolbachia* strains, wMel, did not affect any of the studied traits. Another strain, wMelPlus, had a significant effect on the development time. Moreover, this effect is observed not only in the line in which it was discovered but also in the one it was transferred to. When transferred to a new line, wMelPlus also caused changes in survival under heat stress. Thus, it could be concluded that *Wolbachia*–*Drosophila* interaction depends on the genotypes of both the host and the symbiont, but some *Wolbachia* effects could depend not on the genotypes, but on the fact of recent transfer of the symbiont.

## 1. Introduction

Maternally inherited alpha-proteobacterium *Wolbachia pipientis*, best known for its ability to induce cytoplasmic incompatibility and manipulate host reproduction [1], occurs in more than 40% of arthropod species, including *Drosophila melanogaster* [2,3]. According to its effect on reproductive biology of the host, *Wolbachia* has long been considered a parasite, but now a lot of data have been accumulated indicating that the host, in turn, can benefit from *Wolbachia* infection [4]. 

Another positive aspect of this symbiosis is the possibility of using *Wolbachia* in the control of insect pests. *Wolbachia* have two main uses in this regard: incompatible insect technique (IIT) based on cytoplasmic incompatibility (CI) caused by *Wolbachia* in many insect species [5], and pathogen blocking technique (PBT) based on the ability of *Wolbachia* to provide antiviral protection to their host and to spread in a wild population due to CI-provided fitness advantages of *Wolbachia*-infected females [6,7]. IIT achieves a suppression of insect pests’ populations due to *Wolbachia*-infected males’ failure to produce viable embryos by mating with wild-type uninfected females [8]. PBT blocks the spread of distinct human pathogens, including Zika, Dengue, Yellow fever and West Nile viruses, the Plasmodium parasites that cause malaria, and filarial worms in insect vectors such as *Aedes aegypti* and *Ae. albopictus* [7,9]. The combination of IIT and female sterilization with ionizing radiation was recently used for the population suppression of a fruit pest, *Drosophila suzukii* [10,11], making *D. melanogaster* a valid model for *Wolbachia* studies.

In the members of the *Drosophila* genus, some evidence concerning the role of the genetic features of both bacterium and its host in their interaction was obtained. The antiviral protection of *Wolbachia* strains transferred to the same genetic background of *D. simulans* from different *Drosophila* species depends on the *Wolbachia* genotype [12]. Various *Wolbachia* genotypes transferred to the same genetic background of *D. melanogaster* also demonstrate different effects on the host’s hormonal status and survival under heat stress [13,14]. On the other hand, the positive effect of *Wolbachia* infection on *D. melanogaster* longevity [15] as well as fitness benefits caused by a *Wolbachia* infection in population cage experiments in *D. simulans* [16] depend on the fly genotype.

In *D. melanogaster*, *Wolbachia* genotypes are classified into two groups, wMel and wMelCS, based on polymorphic markers [17,18,19]. The wMel group is dominant all over the world and thus could be considered as giving more benefits to its host [17,20]. However, we have earlier discovered a *Wolbachia* strain, wMelPlus, which belongs to the wMelCS group of genotypes but differs from other described members of this group by a large inversion [21], and found out that this strain changes host fertility [14] and heat stress resistance [22]. The positive effect of the wMelPlus strain on *D. melanogaster* survival under acute heat stress was demonstrated on two different lines, Bi90^T^ and Canton S, after infection transfer from donor w153 line to them by 10–20 generations of backcross with corresponding males [22]. On the other hand, the effect of wMelPlus *Wolbachia* on its “native” host, *D. melanogaster* line w153, was never studied. At the same time, the uninfected Bi90^T^ line obtained by tetracycline treatment from wild type line Bi90, which carried wMel *Wolbachia* from the beginning, did not differ from it in heat stress resistance and fertility level [13]. 

So we could not be certain if the previously discovered effects of wMelPlus *Wolbachia* on the host’s fitness depends only on the strain’s characteristics and not on the effect of *Wolbachia* transfer from line to line. In order to clarify this question, we performed the present study of fertility level, developmental rate, and stress resistance in two pairs of *D. melanogaster* lines: Bi90–Bi90^T^ and w153–w153^T^, infected and uninfected with wMel and wMelPlus *Wolbachia* genotypes, respectively. The characteristics of line Bi90^wMelPlus^ carrying the wMelPlus strain on the Bi90^T^ line’s genetic background were also investigated when the effect of the strain on the trait under study was found in the “native” host line, w153, compared to the tetracycline-treated w153^T^ line.

## 2. Materials and Methods

### 2.1. Drosophila Lines and Rearing

The females of the following *D. melanogaster* lines were used in the study: isofemale lines w153, carrying *Wolbachia* infection of wMelPlus genotype, and Bi90 carrying infection of wMel genotype, established as full-sib families from a single inseminated wild-caught female from Tashkent (Uzbekistan) and Bishkek (Kyrgystan), correspondingly [19]; the corresponding uninfected lines, w153^T^ and Bi90^T^, treated with tetracycline for three generations no less than 10 generations prior to the start of the experiments; and line Bi90^wMelPlus,^ obtained as a result of wMelPlus strain transfer to the Bi90^T^ line by backcrossing with Bi90^T^ males for 20 generations as described earlier [22].

Flies were maintained on standard food (agar-agar, 7 g/L; corn grits, 50 g/L; dry yeast, 18 g/L; sugar, 40 g/L) in a MIR-554 incubator (Sanyo, Osaka, Japan) at 25 °C under a 12:12 h light–dark cycle.

### 2.2. Developmental Rate Analysis

Males and females with a difference in age no more than 4–5 h since eclosion were chosen as parents; as they reached 3 days of age, flies were placed into vials (3–5 parent pairs; 10 vials per experiment group), where they laid eggs for 24 h. Eclosed progeny (imagoes) were counted every 12 h, at 9 a.m. and 9 p.m., up to the eclosion of the last descendant. Developmental rate was presented as a percentage of the total number of eclosed progeny for every period of measurement.

### 2.3. Fertility Analysis

To measure fertility, 3 or 5 pairs of young females and males with a difference in age no more than 4–5 h since eclosion were placed into vials (10 vials per experiment group), where they laid eggs under standard conditions. For 10 days, the flies were placed into new vials every 24 h for oviposition. Fertility was measured as the number of progeny (imagoes) eclosed from the eggs laid every 24 h per female.

### 2.4. Viability Analysis

To estimate viability under acute heat stress, the vials with flies of all groups (10–15 vials with 5 females and 5 males each per group) at the age of 6 days were transferred from 25 °C to 38 °C for 4 h. Then they were returned to 25 °C and the surviving females were counted 24 h later. The survival rates were calculated as the percentage of survivors in each vial. 

### 2.5. Statistical Analyses 

#### 2.5.1. Fertility 

Each culture vial was considered as a separate case, and the fertility in it per female per day was considered as a separate trait. Euclidean distance was used to estimate differences between vials by fertility over a range of days. The matrix of pairwise Euclidean distances by fertility between all vials of all considered lines was processed by the principal coordinate method (PCoA). For Euclidean distances, this method is equivalent to principal component analysis (PCA) [23]. As a rule, differences between lines manifested themselves in one of the first two principal components, which together accounted for more than half of the total variance. The significance of the differences between each pair of lines for each principal component was assessed using a two-sample *t*-test, applying the Benjamini–Hochberg P adjustment to correct for multiple testing [24].

Some inconvenience of the Benjamini–Hochberg P adjustment is that it is necessary, in addition to each sample’s p_i_-value, to calculate the corresponding standard pBH_i_-adjustment for comparison. However, the calculation can be greatly simplified by multiplying both indicators by N/i. Then the indicator Np_i_ = p_i_-value × N/i must be compared with NpBH_i_ = pBH_i_-adjustment × N/i = iα/N× N/i = α, that is, with the standard tabular level to which everyone is accustomed. It is simple, but very convenient. For i = 1, this technique also works for the Bonferroni method [25].

For convenience of calculation and presentation of results, each pair of lines compared by one quantitative characteristic (for example, by the principal component) was combined into one sample and a dichotomous variable was additionally formed for it so that each value of the quantitative sample was marked 0 for one of the lines, 1 for another. The Pearson correlation coefficient r was calculated between the quantitative variable and the dichotomous one. It is known [26] that calculating the significance of this point-biserial correlation coefficient r is equivalent to calculating the significance of Student’s *t*-test used to compare the means of two normal populations with equal variance. Additionally, the squared correlation coefficient (r^2^) is an estimate of the currently recommended effect size [27], so we also present it in the tables.

#### 2.5.2. Developmental Rate

Every 12 h, the number of emerging flies was recorded for each vial. After the end of the experiment, the resulting dynamics were normalized to the total number of all eclosed flies. Euclidean distance was used to assess differences in developmental rates for each pair of vials. Next, just as for fertility, the principal components were calculated by the Gower principal coordinate method and the lines were compared with each other according to the first two principal components by *t*-test, applying the Benjamini–Hochberg P adjustment [24] (see the previous section).

#### 2.5.3. Viability

Survival rates were calculated as the percentage of survivors in each vial. The groups were compared on this trait using the *t*-test by the Benjamini-Hochberg method [24]. 

## 3. Results

### 3.1. Fertility of D. melanogaster Lines w153 and Bi90 Infected with the wMelPlus and wMel Wolbachia Strains, Correspondingly, and Control Uninfected Lines w153^T^ and Bi90^T^

**Figure 1:**
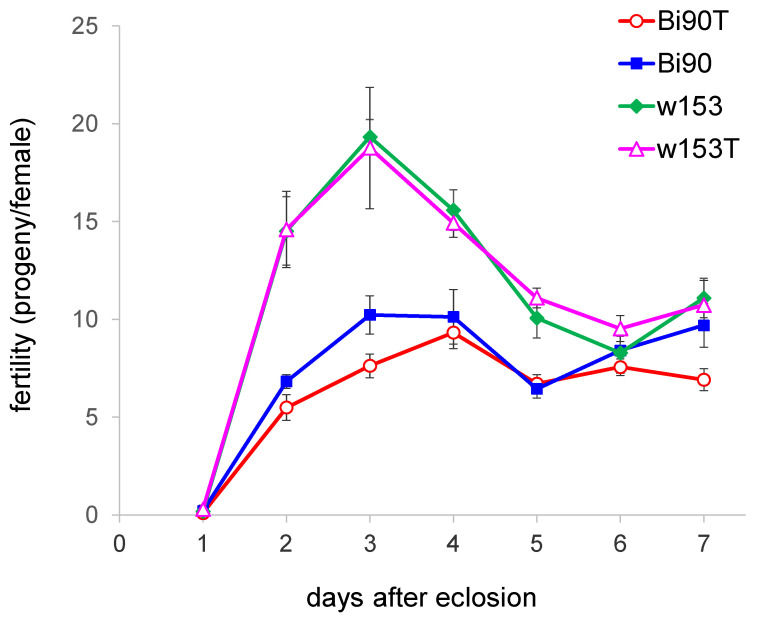
The fertility of *D. melanogaster* wild type lines Bi90 (infected with wMel *Wolbachia* strain), w153 (infected with wMelPlus *Wolbachia* strain), Bi90^T^ (uninfected), w153^T^ (uninfected). Each point represents an average value of 9–10 replicates (3 females per test) as means ± s.e.m.

Earlier, we had shown that the wMelPlus strain of *Wolbachia* being transferred to Bi90^T^ line of *D. melanogaster* caused significant changes in host fertility level [14], so the first thing to study was the fertility of the “native” wMelPlus line w153 in comparison with the uninfected (tetracycline-treated) line w153^T^ (Figure 1). 

**Figure 2:**
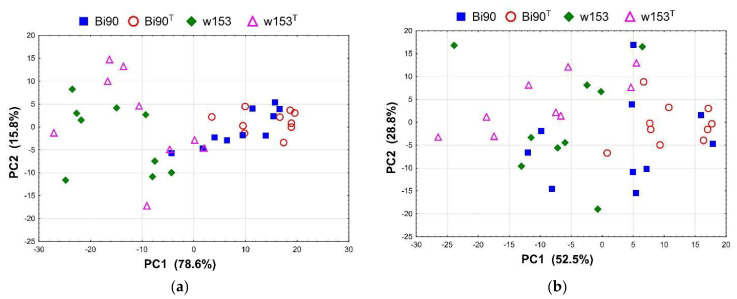
PCA plot showing variability of the fertility level per female per day in the Bi90 (infected with wMel *Wolbachia* strain), w153 (infected with wMelPlus *Wolbachia* strain), Bi90^T^ (uninfected), w153^T^ (uninfected) lines of *D. melanogaster*: (**a**) days 2–4 after eclosion; (**b**) days 5–7 after eclosion. Each data point represents one biological replicate (three females per replicate).

The Bi90^T^ line, together with its precursor, the Bi90 line naturally infected with the wMel strain, were taken into analysis as well (Figure 1). Analysis of the fertility curve shows that, according to the level of fertility, this period can be divided into 2 sub-periods: (1) typical fertility peak reached between 2 and 4 days after eclosion [28] and (2) subsequent decrease in fertility level from 5 to 7 days after eclosion. In the first sub-period, lines with different genetic backgrounds (originating from the Bi90 line and originating from the w153 line) strongly differ from each other. In the second sub-period, the differences are smoothed out. We analyzed the differences between all four lines in these sub-periods using the principal component method (Figure 2).

In the first sub-period, lines Bi90 and Bi90^T^ clearly differ from lines w153 and w153^T^, implying differences in fertility caused by the genetic component of the host, i.e., by *D. melanogaster* genotype (Figure 2a). However, there are no differences observed between both the wMel-infected Bi90 line and the uninfected line Bi90^T^, and between the wMelPlus-infected w153 line and the uninfected line w153^T^. In other words, component analysis did not reveal any differences in fertility for this period resulting from the presence/absence of both *Wolbachia* strains under study.

Statistical assessment of the fertility level for days 2–4 is given in Tables S1 and S2, presenting a pairwise comparison of all lines under study using the *t*-test (below the diagonal) by the Benjamini–Hochberg method (above the diagonal). There is a high significance level for PC1 of the differences between the lines of Bi90 genotype compared with the lines of w153 genotype (Table S1); there are no significant differences for PC2 (Table S2).

The results of comparing fertility levels of the Bi90 (infected with wMel *Wolbachia* strain), w153 (infected with wMelPlus *Wolbachia* strain), Bi90^T^ (uninfected), and w153^T^ (uninfected) lines in the second sub-period (days 5–7) by the principal component method are presented in Figure 2b. Significant differences between lines can be seen, just as in the first sub-period. However, they are less prominent, which is evidenced by minimal (but significant) *t*-test by the Benjamini–Hochberg method for PC1 (Table S3). For PC2, differences are insignificant (Table S4).

### 3.2. Developmental Rate of D. melanogaster Lines w153 and Bi90 Infected with the wMelPlus and wMel Wolbachia Strains, Correspondingly, and Control Uninfected Lines w153^T^ and Bi90^T^

**Figure 3:**
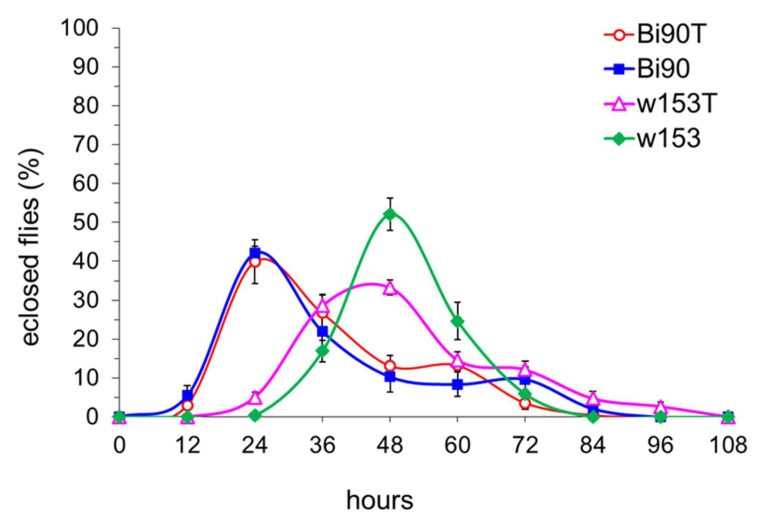
The developmental rate of *D. melanogaster* wild type lines Bi90 (infected with wMel *Wolbachia* strain), w153 (infected with wMelPlus *Wolbachia* strain), Bi90^T^ (uninfected), w153^T^ (uninfected). Each point represents the percentage of flies eclosed during 12 h (9–10 biological replicates per point; five flies in each replicate, means ± s.e.m.

Working with the Bi90 and w153 *D. melanogaster* lines infected with wMel andwMelPlus *Wolbachia* strains, correspondingly, we have noted that eclosion in the w153 line occurs one day later than in the Bi90 line. For this reason, we analyzed the developmental rates of these two lines as well as their uninfected versions, Bi90^T^ and w153^T^. Figure 3 presents developmental rate curves expressed as a percentage of the number of eclosed flies: from the first to the last, counted at equal 12-h intervals. In the wMel-infected Bi90 line and the uninfected Bi90^T^ line, developmental rate curves almost match; in the wMelPlus-infected w153 line, the eclosion peak is delayed by 24 h compared to the Bi90 and Bi90^T^ lines, and the peak in the uninfected line w153^T^ is close to that of w153. 

**Figure 4:**
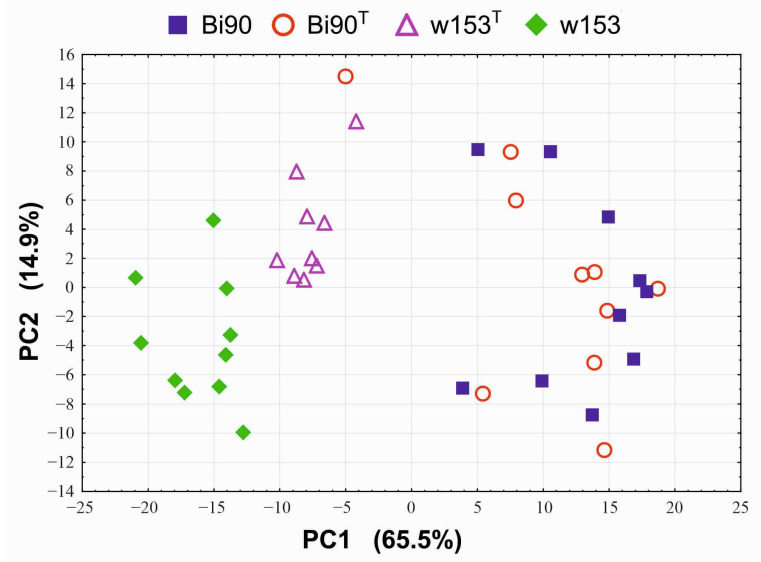
PCA plot showing the variability of developmental rate in the Bi90 (infected with wMel *Wolbachia* strain), w153 (infected with wMelPlus *Wolbachia* strain), Bi90^T^ (uninfected), w153^T^ (uninfected) lines of *D. melanogaster*: Each point (one replicate) represents the percentage of flies eclosed during 12 h (five flies in each replicate).

Developmental rate analysis by the principal component method shows that while lines with the same genetic background (Bi90 and Bi90^T^) do not differ from each other, they differ by PC1 from lines with a different genetic background (w153 and w153^T^) with a high significance level (Figure 4).

Tables S5 and S6 show *t*-test by the Benjamini–Hochberg method for PC1 and PC2, correspondingly. The significance is very high: for NpBH, it is exponential. Notably, developmental rates also significantly differ between lines w153 and w153^T^ both by PC1 (Table S5) and by PC2 (Table S6), which may imply that the *Wolbachia* strain wMelPlus influences the developmental rate of the w153 line.

### 3.3. Developmental Rate of the D. melanogaster Line Bi90 Infected with the wMelPlus Wolbachia Strain in Comparison with the Control Uninfected Line Bi90^T^

**Figure 5:**
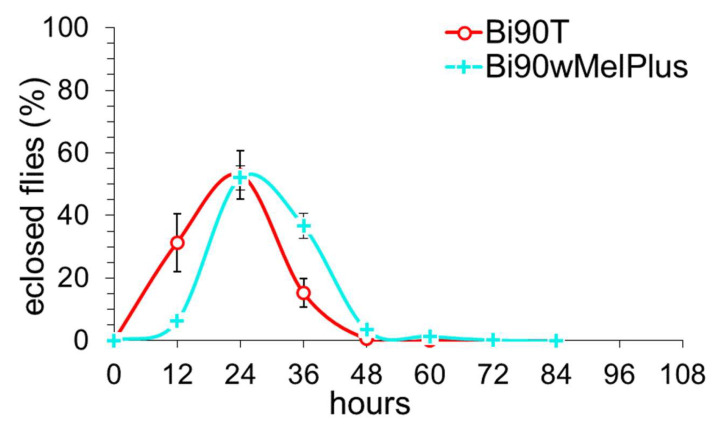
The developmental rate of *D. melanogaster* uninfected line Bi90^T^ and line Bi90^wMelPlus^, carrying *Wolbachia* strain wMelPlus from line w153 on nuclear background of line Bi90^T^. Each point represents the percentage of flies eclosed during 12 h (10 biological replicates per line; five flies in each replicate) as means ± s.e.m.

In order to verify our assumption concerning the effect of the wMelPlus strain on host developmental rate, we studied this trait in the Bi90^wMelPlus^ line, which carries wMelPlus *Wolbachia* on Bi90^T^ nuclear background, in comparison with the uninfected line Bi90^T^ (Figure 5).

**Figure 6:**
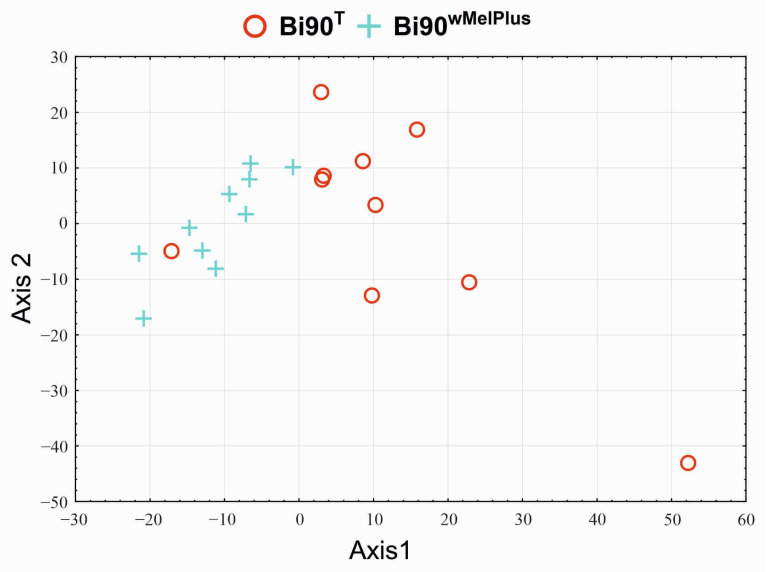
PCA plot showing the variability of developmental rate in *D. melanogaster* uninfected line Bi90^T^ and line Bi90^wMelPlus^, carrying *Wolbachia* strain wMelPlus from line w153 on nuclear background of line Bi90^T^. Each point represents the percentage of flies eclosed during 12 h (one biological replicates per point; five flies in each replicate).

The PCA plot (Figure 6) allowed us to determine that the differences in developmental rate between the Bi90^wMelPlus^ and Bi90^T^ lines are significant in both PC1 and PC2 (Table S7); or significant at t = 3.73, NpBH = 0.00168 if we rotate the PCA plot by 30° (Figure S1).

### 3.4. Survival under Acute Heat Stress of D. melanogaster Lines w153 and Bi90 Infected with the wMelPlus and wMel Wolbachia Strains, Correspondingly, and the Bi90 Line Infected with the wMelPlus Strain in Comparrison with Control Uninfected Lines Bi90^T^ and w153^T^

**Figure 7:**
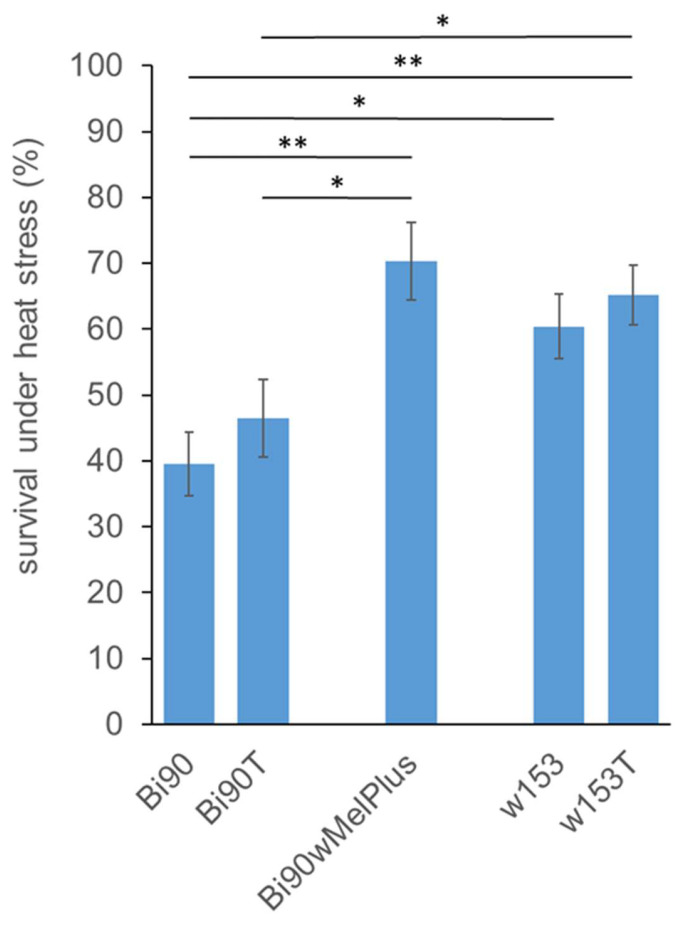
Survival under heat stress (38 °C, 4 h) of *D. melanogaster* females of lines Bi90 (infected with wMel *Wolbachia* strain), w153 (infected with wMelPlus *Wolbachia* strain), Bi90^T^ (uninfected), w153^T^ (uninfected). Each point represents an average value of 30–35 tests (N = 4 or 5 for each test) as means ± s.e.m. The asterisk indicates significant differences between the lines (**, *p* < 0.01; *, *p* < 0.05).

Earlier, we showed that the Bi90^wMelPlus^ line is characterized by increased viability under acute heat stress compared with the Bi90 lines infected with other *Wolbachia* strains [22], so it was of interest to find out if the wMelPlus strain causes the same effect in its “native” host, line w153. The analysis of survival under heat stress (38 °C, 4 h) carried out in the Bi90, Bi90^T^, Bi90^wMelPlus^, w153 and w153^T^ lines demonstrated that the line pairs Bi90–Bi90^T^ and w153–w153^T^ did not differ in stress resistance within the pairs (Figure 7, Table S8). 

However, survival rate under heat stress in both lines with nuclear background of the wMelPlus-infected w153 line was significantly higher than that of the wMel-infected Bi90 line, and w153^T^’s survival rate was higher than that of Bi90^T^ (Figure 7, Table S8), which suggests a strong effect of the *D. melanogaster* genotype on the trait under study. On the other hand, the maximum survival rate was demonstrated by the Bi90^wMelPlus^ line differing significantly from the Bi90 and Bi90^T^ lines (Figure 7, Table S8), which could be evidence of *Wolbachia* genotype input to the trait value. At the same time, it should be noted that there was no significant difference in stress resistance between the Bi90^wMelPlus^ line and the w153 and w153^T^ lines (Figure 7, Table S8). 

## 4. Discussion

Most studies performed on *D. melanogaster* have used only a few “wild-type” strains, representing very little genetic diversity. However, it is the genetic variation that is one of the main drivers of the evolution of life-history traits. Major life-history traits, which are subject to evolution by natural selection and are therefore vital to understanding adaptation, include developmental rate, size at eclosion, progeny number, life span, and various stress resistance traits [28]. Here, we present data on two wild-caught isofemale lines demonstrating significant differences in three life-history traits, namely, developmental rate, fertility (estimated as progeny per female), and survival under acute heat stress. We found out that lines Bi90^T^ and w153^T^ differ significantly in all traits under study. It was shown that fertility level could be highly variable among both laboratory lines and wild populations measured in the laboratory [29,30,31] but correlations between progeny number and developmental rate were usually positive [28]. However, females of the w153^T^ line demonstrated a higher fertility level and a lower developmental rate compared to the Bi90^T^ line (see Figure 1, Figure 2a, Figure 3 and Figure 4), i.e., these two traits were negatively correlated, which was rather unexpected for us. Moreover, reduced fertility observed in the Bi90^T^ line (compared to the w153^T^ line) was shown to correlate to increased resistance to such types of stressors as desiccation and starvation [32,33], but in our experiments it went together with decreased resistance to heat stress (see Figure 7). This could mean the existence of a different mechanism of resistance to different types of stress, the specificity of lines under study, or even both. The fact that stands for the uniqueness of one of the studied lines, w153^T^, is that it carries the rather unique *Wolbachia* strain wMelPlus, which not only alters its “native” host’s developmental rate (see Figure 3 and Figure 4), but also changes the fertility level, starvation, and heat stress resistance of the host when transferred to a new *D. melanogaster* line [14,22,34]. It should be noted that for other *Wolbachia* strains (except the well-known pathogenic strain wMelPop [35]) no such effects were found either in the “native” host or following transfer to a new one [13,14,22].

There is evidence that the interaction between *Wolbachia* and *Drosophila* has a complex nature. For example, it was found that a single *Wolbachia* strain wHa being transferred into three genetically distinct isofemale lines of *Drosophila simulans* with the use of microinjection methodology caused a dramatic fitness benefit in one of these lines and did not affect the fitness of two others [16]. The transfection of a single *Wolbachia* strain of wMel genotype into two different *Drosophila* species, *D. melanogaster* and *D. nigrosparsa*, resulted in completely different changes in the differential expression of genes [36]. 

On the other hand, different *Wolbachia* strains being transferred to the same *D. melanogaster* line Bi90^T^ caused different effects on fertility level, dopamine metabolism, and resistance to heat stress [13,14,22]. The results obtained in the present paper correspond with these data: the wMelPlus strain, which belongs to the wMelCS *Wolbachia* genotype and is shown to affect the fertility of females of line Bi90^wMelCS^ [14], does not influence this trait in its “native” host, the *D. melanogaster* line w153 (see Figure 1 and Figure 2, Tables S1 and S2). Similarly, the wMelPlus strain increases resistance to acute heat stress when transferred to the Canton S or Bi90^T^ lines [22], but does not affect it in w153 (see Figure 7). 

Several attempts to shed light on the molecular mechanisms of the effect of *Wolbachia* on host’s physiology have been made. The transcriptome analyses of infected *D. melanogaster* females performed recently demonstrated changes in differential gene expression, which allowed to relate them to the Gene Ontology terms *Iron ion binding* and *Oxidation–reduction process* [36] or to create protein–protein interaction networks in STRING with the strongest interactions including *Metabolism*, *Ubiquitin*, *RNA binding and processing*, *Transcription and translation* and *Stress* [37]. The latter correlates with our findings concerning increased stress resistance of wMelPlus-infected females, and data on changes in metabolism correspond with results on increased lipid and glucose content found in both Bi90 and Bi90^wMelPlus^ females [34]. However, it is not obvious how the findings made in transcriptome analyses are connected with the effect of *Wolbachia* on fertility or developmental rate.

It is well-known that effects of many mutations found in one genetic background are often suppressed or enhanced in other backgrounds [38]. According to the data obtained here and presented in other investigations, it seems possible to suggest that an epistatic interaction of this kind could be discovered in genetic interaction between a host and a symbiotic bacterium, *Wolbachia* in particular. 

However, not all effects of the wMelPlus strain depend on the host genotype: one can see that it slows down developmental rate and postpones eclosion in both w153 and Bi90^wMelCS^ lines (see Figure 3, Figure 4, Figure 5 and Figure 6). It was shown in the end of the last century and the beginning of the present one that considerable variation in egg-to-adult development time could occur among wild-type strains of various *Drosophila* species [39,40]. As testing flies for the presence of *Wolbachia* was not common practice at the time, and *Drosophila* has been shown to have high rates of infection [2], one cannot be sure that at least part of the variables observed in these studies were not caused by *Wolbachia*. Another possible explanation for our findings could be the uniqueness of the wMelPlus *Wolbachia* strain, which is the only one to be found to increase resistance to acute heat stress [14,22] and to change the host’s developmental rate (see Figure 3, Figure 4, Figure 5 and Figure 6). The latter assumption is indirectly confirmed by the data of Strunov et al. [41], who found that the wMelCS type of infection and the wMel type did not influence any developmental life-history traits. 

It should be noted that the results which demonstrate that wMelCS-infected flies were more fertile than wMel-infected flies, while the latter did not differ in fertility from uninfected flies [41], also agree with our data showing that the wMel *Wolbachia* strain, which infected the Bi90 line, does not cause any effects on the life-history traits under study. No changes in fertility level, developmental rate, and stress resistance in the Bi90 line compared to the uninfected Bi90^T^ line were observed, while the w153 line infected with *Wolbachia* of the wMelCS type has increased early life fertility level compared to the Bi90 and Bi90^T^ lines (Figure 1, Figure 2, Figure 3, Figure 4, Figure 5, Figure 6 and Figure 7). Increased usefulness of the wMelCS type of *Wolbachia* compared to the wMel type in terms of enhancing the host’s fertility was also demonstrated in the experiments with fertility rescue in flies with the *bag of marbles* (*bam*) hypomorphic mutation [42]. Moreover, wMelCS-like *Wolbachia* variants were shown to provide stronger protection against *Drosophila* Flock House and C viruses compared to wMel-like variants as well [43]. 

Thus, it could be concluded that *Wolbachia*–*Drosophila* interaction depends on the genotypes of both the host and the symbiont. However, taking into account that some of wMelPlus effects on life-history traits occur in the infected Bi90 and Canton S lines and not in the “native” line for this strain, w153, it could be hypothesized that at least some of the effects which occur in a *D. melanogaster* host infected with *Wolbachia* depend not on the genotype of the symbiont, but on the fact of its recent transfer. And one more supposition is possible as the w153^T^ line is characterized by increased early life fertility and stress resistance (see Figure 1, Figure 2 and Figure 7) even in the absence of the wMelPlus *Wolbachia* strain (see Figure 1, Figure 2 and Figure 7). We suppose that it could be an evidence of a successful co-evolution of the host line w153 and the symbiont *Wolbachia* strain wMelPlus. It can also be said that our data provides some insight into the prospects for the use of *Wolbachia* in pest control, indicating the need for thorough genetic studies of *Wolbachia* strains in pest species, such as *D. suzuki* or mosquitoes of the *Aedes* genus, aimed at finding the genetic variations of the bacterium most suitable for IIT and PBT. 

## Data Availability

Data are contained within the article.

## Supplementary Materials

The following supporting information can be downloaded at: https://www.mdpi.com/article/10.3390/insects14120928/s1, Table S1. Significance level for PC1 of the fertility differences between the Bi90 (infected with wMel *Wolbachia* strain), w153 (infected with wMelPlus strain), Bi90^T^ (uninfected), w153^T^ (uninfected) lines for days 2–4 after eclosion using the *t*-test by the Benjamini–Hochberg method. Table S2. Significance level for PC2 of the fertility differences between the Bi90 (infected with wMel *Wolbachia* strain), w153 (infected with wMelPlus strain), Bi90^T^ (uninfected), w153^T^ (uninfected) lines for days 2–4 after eclosion using the *t*-test by the Benjamini–Hochberg method. Table S3. Significance level for PC1 of the fertility differences between the Bi90 (infected with wMel *Wolbachia* strain), w153 (infected with wMelPlus strain), Bi90^T^ (uninfected), w153^T^ (uninfected) lines for days 5–7 after eclosion using the *t*-test by the Benjamini–Hochberg method. Table S4. Significance level for PC2 of the fertility differences between Bi90 (infected with wMel *Wolbachia* strain), w153 (infected with wMelPlus strain), Bi90^T^ (uninfected), w153^T^ (uninfected) lines for days 5–7 after eclosion using the *t*-test by the Benjamini–Hochberg method. Table S5. Significance level for PC1 of the differences in developmental rate between the Bi90 (infected with wMel *Wolbachia* strain), w153 (infected with wMelPlus strain), Bi90^T^ (uninfected), w153^T^ (uninfected) lines with the *t*-test by the Benjamini–Hochberg method. Table S6. Significance level for PC2 of the differences in developmental rate between the Bi90 (infected with wMel *Wolbachia* strain), w153 (infected with wMelPlus strain), Bi90^T^ (uninfected), w153^T^ (uninfected) lines using the *t*-test by the Benjamini–Hochberg method. Table S7. Significance level for PC1 and PC2 of the differences in developmental rate between the Bi90^T^ and Bi90^wMelPlus^ lines using the *t*-test by the Benjamini–Hochberg method. Figure S1. PCA plot (after a 30° rotation) showing the variability of developmental rate in the Bi90^T^ and Bi90^wMelPlus^ lines of *D. melanogaster*. Each point represents the percentage of flies eclosed during 12 h (one biological replicates per point; five flies in each replicate). Table S8. Significance level of the differences in survival under acute heat stress between the Bi90 (infected with wMel *Wolbachia* strain), w153 (infected with wMelPlus *Wolbachia* strain), Bi90^T^ (uninfected), w153^T^ (uninfected) lines using the *t*-test by the Benjamini–Hochberg method.
