# Supplementary material for: The Effect of Genotype Combinations of Wolbachia and Its Drosophila melanogaster Host on Fertility, Developmental Rate and Heat Stress Resistance of Flies
Source: Insects. 2023 Dec 5;14(12):928. doi: 10.3390/insects14120928 (PMC10743879; doi:10.3390/insects14120928)
Supplement: Supplementary file 1 [file insects-14-00928-s001.zip › insects-2713044-supplementary.pdf]

Table S1. Significance level for PC1 of the fertility differences between the Bi90 (infected with wMel *Wolbachia* strain), w153 (infected with wMelPlus strain), $Bi90^T$ (uninfected), $w153^T$ (uninfected) lines for days 2-4 after eclosion using the *t*-test by the Benjamini–Hochberg method.

t \ NpBH	Bi90	$Bi90^T$	w153	$w153^T$
**Bi90**		0.096543	5.84E-06	0.0001024
**$Bi90^T$**	1.86		4.39E-07	5.57E-06
**w153**	7.02	9.31		0.278902
**$w153^T$**	5.33	7.28	1.12	

Each p-value was multiplied by (N/i) and compared to generally accepted reference values of 0.05, 0.01 and 0.001.

Table S2. Significance level for PC2 of the fertility differences between the Bi90 (infected with wMel *Wolbachia* strain), w153 (infected with wMelPlus strain), $Bi90^T$ (uninfected), $w153^T$ (uninfected) lines for days 2-4 after eclosion using the *t*-test by the Benjamini–Hochberg method.

t \ NpBH	Bi90	$Bi90^T$	w153	$w153^T$
**Bi90**		0.925	0.744	0.771
**$Bi90^T$**	1.05		1.158	0.970
**w153**	0.70	1.36		0.833
**$w153^T$**	0.47	0.04	0.84	

Each p-value was multiplied by (N/i) and compared to generally accepted reference values of 0.05, 0.01 and 0.001.

Table S3. Significance level for PC1 of the fertility differences between the Bi90 (infected with wMel *Wolbachia* strain), w153 (infected with wMelPlus strain), $Bi90^T$ (uninfected), $w153^T$ (uninfected) lines for days 5-7 after eclosion using the *t*-test by the Benjamini–Hochberg method.

t \ NpBH	Bi90	$Bi90^T$	w153	$w153^T$
**Bi90**		0.0666	0.0537	0.0380
**$Bi90^T$**	2.17		0.0003	0.0004
**w153**	2.18	5.21		0.5466
**$w153^T$**	2.61	5.33	0.62	

Each p-value was multiplied by (N/i) and compared to generally accepted reference values of 0.05, 0.01 and 0.001.

Table S4. Significance level for PC2 of the fertility differences between Bi90 (infected with wMel *Wolbachia* strain), w153 (infected with wMelPlus strain), $Bi90^T$ (uninfected), $w153^T$ (uninfected) lines for days 5-7 after eclosion using the *t*-test by the Benjamini–Hochberg method.

t \ NpBH	Bi90	$Bi90^T$	w153	$w153^T$
**Bi90**		0.575	0.526	0.235
**$Bi90^T$**	1.10		0.794	0.204
**w153**	0.96	0.26		0.512
**$w153^T$**	2.25	1.96	0.82	

Each p-value was multiplied by (N/i) and compared to generally accepted reference values of 0.05, 0.01 and 0.001.

Table S5. Significance level for PC1 of the differences in developmental rate between the Bi90 (infected with wMel *Wolbachia* strain), w153 (infected with wMelPlus strain), $Bi90^T$ (uninfected), $w153^T$ (uninfected) lines with the t-test by the Benjamini–Hochberg method.

**t \ NpBH**	**Bi90**	**$Bi90^T$**	**w153**	**$w153^T$**
**Bi90**		0.4435	1.04E-10	7.94E-09
**$Bi90^T$**	0.78		6.71E-09	1.12E-06
**w153**	15.55	11.39		1.38E-06
**$w153^T$**	11.47	7.82	7.56	

Each p-value was multiplied by (N/i) and compared to generally accepted reference values of 0.05, 0.01 and 0.001.

Table S6. Significance level for PC2 of the differences in developmental rate between the Bi90 (infected with wMel *Wolbachia* strain), w153 (infected with wMelPlus strain), $Bi90^T$ (uninfected), $w153^T$ (uninfected) lines using the *t*-test by the Benjamini–Hochberg method.

**t \ NpBH**	**Bi90**	**$Bi90^T$**	**w153**	**$w153^T$**
**Bi90**		4.352	1.317	0.565
**$Bi90^T$**	0.36		0.845	1.578
**w153**	1.27	1.54		0.00513
**$w153^T$**	1.78	1.16	4.09	

Each p-value was multiplied by (N/i) and compared to generally accepted reference values of 0.05, 0.01 and 0.001.

Table S7. Significance level for PC1 and PC2 of the differences in developmental rate between the $Bi90^T$ and $Bi90^{wMelPlus}$ lines using the *t*-test by the Benjamini–Hochberg method.

	**t**	**NpBH**
**PC1**	2.63	0.018
**PC2**	2.19	0.043

Each p-value was multiplied by (N/i) and compared to generally accepted reference values of 0.05, 0.01 and 0.001.

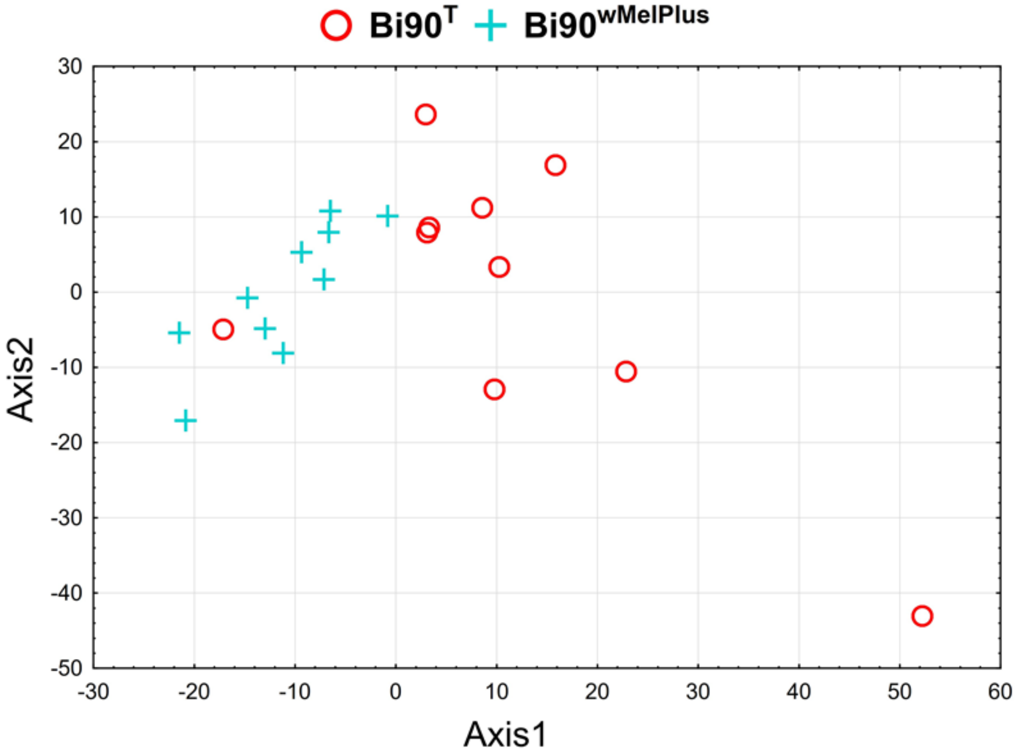

Figure S1. PCA plot (after a 30° rotation) showing the variability of developmental rate in the Bi90^T and Bi90^{wMelPlus} lines of *D. melanogaster*. Each point represents the percentage of flies eclosed during 12 hours (one biological replicates per point; five flies in each replicate).

Table S8. Significance level of the differences in survival under acute heat stress between the Bi90 (infected with wMel *Wolbachia* strain), w153 (infected with wMelPlus *Wolbachia* strain), Bi90^T (uninfected), w153^T (uninfected) lines using the *t*-test by the Benjamini–Hochberg method.

t \ NpBH	Bi90	Bi90^T	Bi90^{wMelPlus}	w153	w153^T
**Bi90**		0.439	0.002	0.011	0.001
**Bi90^T**	0.94		0.013	0.112	0.025
**Bi90^{wMelPlus}**	4.04	2.90		0.281	0.495
**w153**	3.06	1.86	1.30		0.533
**w153^T**	3.97	2.58	0.69	0.71	

Each p-value was multiplied by (N/i) and compared to generally accepted reference values of 0.05, 0.01 and 0.001.
